# Cardiac Arrhythmias in Survivors of Sudden Cardiac Death Requiring Impella Assist Device Therapy

**DOI:** 10.3390/jcm10071393

**Published:** 2021-03-31

**Authors:** Khaled Q. A. Abdullah, Jana V. Roedler, Juergen vom Dahl, Istvan Szendey, Dimitrios Dimitroulis, Lars Eckardt, Albert Topf, Bernhard Ohnewein, Lorenz Fritsch, Fabian Föttinger, Mathias C. Brandt, Bernhard Wernly, Lukas J. Motloch, Robert Larbig

**Affiliations:** 1 Department of Cardiology, Hospital Maria Hilf Moenchengladbach, 41063 Moenchengladbach, Germany; KhaledQaidAhmed.Abdullah@mariahilf.de (K.Q.A.A.); janaviennarose@icloud.com (J.V.R.); Juergen.Dahlvom@mariahilf.de (J.v.D.); Istvan.Szendey@mariahilf.de (I.S.); Dimitrios.Dimitroulis@mariahilf.de (D.D.); 2 Department of Cardiology, University Hospital Aachen, RWTH University Aachen, 52062 Aachen, Germany; 3 Department of Cardiovascular Medicine, Division of Electrophysiology, University of Muenster, 48149 Muenster, Germany; lars.eckardt@ukmuenster.de; 4 Department of Cardiology, Clinic II for Internal Medicine, University Hospital Salzburg, Paracelsus Medical University, 5020 Salzburg, Austria; albert.topf@hotmail.com (A.T.); b.ohnewein@salk.at (B.O.); l.fritsch@salk.at (L.F.); Fabian.Foettinger@stud.pmu.ac.at (F.F.); m.brandt@salk.at (M.C.B.); bernhard@wernly.net (B.W.); l.motloch@salk.at (L.J.M.); 5 Department of Anesthesiology, Perioperative Medicine and Intensive Care Medicine, Paracelsus Medical University of Salzburg, 5026 Salzburg, Austria; 6 Center for Public Health and Healthcare Research, Paracelsus Medical University of Salzburg, 5026 Salzburg, Austria

**Keywords:** sudden cardiac death, Impella, assist devices, arrhythmias, asystole, ventricular tachycardia, atrial fibrillation

## Abstract

In this retrospective single-center trial, we analyze 109 consecutive patients (female: 27.5%, median age: 69 years, median left ventricular ejection fraction: 20%) who survived sudden cardiac death (SCD) and needed hemodynamic support from an Impella assist device between 2008 and 2018. Rhythm monitoring is investigated in this population and associations with hospital survival are analyzed. Hospital mortality is high, at 83.5%. Diverse cardiac arrhythmias are frequently registered during Impella treatment. These include atrial fibrillation (AF, 21.1%) and ventricular tachycardia (VT, 18.3%), as well as AV block II°/III° (AVB, 7.3%), while intermittent asystole (ASY) is the most frequently observed arrhythmia (42.2%). Nevertheless, neither ventricular nor supraventricular tachycardias are associated with patients’ survival. In patients who experience intermittent asystole, a trend towards a fatal outcome is noted (*p* = 0.06). Conclusions: Mortality is high in these severely sick patients. While cardiac arrhythmias were frequent, they did not predict hospital mortality in this population. The hemodynamic support of the pump seems to counterbalance the adverse effects of these events.

## 1. Introduction

Sudden cardiac death (SCD) is a leading cause of death in the western world [1,2]. Survivors of SCD are often critically ill and need ventricular assist device support due to systemic hypotension and tissue hypoperfusion. The Impella transvalvular microaxial blood pump (Abiomed, Danvers, USA) is a commonly used assist device in this population for active left ventricular (LV) unloading. The Impella provides intermediate support by increasing cardiac output up to 3–5.0 L/min depending on the generation of the assist device [3,4]. However, mortality is high among survivors of SCD with the need for an assist device, and the therapy with an Impella has a controversial impact on survival [5], while also bearing the potential for sometimes severe complications [6]. Since SCD is caused by the onset of severe cardiac arrhythmias we, therefore, hypothesized that their occurrence throughout the course of treatment, even in the presence of a potent blood pump, could be a predictor of impaired survival. The incidence of cardiac arrhythmias in survivors of SCD with an Impella has not yet been fully characterized. We, therefore, analyzed survivors of SCD treated with an Impella at our hospital with regard to the occurrence and classification of various types of arrhythmias. Additionally, we wanted to analyze the arrhythmias’ individual impact on mortality in this collective.

## 2. Materials and Methods

In this retrospective single-center trial, we analyzed 109 consecutive patients (female: 27.5%, median age: 69 years, median left ventricular ejection fraction: 20%) that survived SCD due to a successful cardiopulmonary resuscitation (CPR). These patients were admitted to our intensive care unit (ICU) from 2008–2018.

We included patients that survived SCD with a previous successful CPR. The majority of our patients presented with acute myocardial infarction (AMI). The indication for Impella support was determined by a systolic blood pressure below 90 mmHg for more than 30 min, a highly decreased left ventricular ejection fraction (LVEF) of less than 30% (determined echocardiographically using eyeballing and the biplane Simpson method in at least two different axes), the presence of elevated serum lactate values > 2 mmol/L or continuous hemodynamic instability despite inotrope or vasopressor therapy. The Impella position was routinely checked every 12 h at our ICU using transthoracic echocardiography performed by a skilled cardiologist and optimized when necessary. Data regarding medication, results from diagnostic tests and history of concomitant diseases and outcomes were obtained from the hospital’s patient database.

In all eligible patients, data were retrospectively collected from electronic medical records. The data obtained comprised demographics, medical history, laboratory examinations, comorbidities, complications, specific treatment measures and outcomes.

Arrhythmia analyses were performed by examination of the continuous single lead telemetry monitoring system of each patient by a skilled physician and experienced ICU nurses. We distinguished between atrial fibrillation (AF), other supraventricular tachycardias (SVTs), higher degree of AV block (second- and third-degree AV block, AVB), sustained ventricular tachycardia (VT) and asystole (ASY), verified by the invasive arterial blood pressure and ECG system in each patient, which was monitored continuously (MetaVision, iMDsoft, Tel Aviv-Yafo, Israel). Possible artifacts were excluded by individual revision of the data. Cardiac arrhythmias during ICU rhythm monitoring were classified according to current guidelines [7]. AF was defined as the presence of an irregular rhythm with fibrillatory waves and no defined P-waves for at least 30 s during rhythm monitoring. Other supraventricular tachycardias were defined as regular rhythm in the absence of P-waves (or appearance of flutter waves) for at least 30 s during rhythm monitoring, consistent with atrial flutter, atrioventricular tachycardia, atrioventricular node tachycardia or focal atrial tachycardia. Ventricular tachycardia was defined as at least four consecutive ventricular beats with non-sustained ventricular tachycardia (nsVT) lasting < 30 s, and sustained ventricular tachycardia lasting ≥ 30 s. Therapy-resistant VT was defined as VT that could not be terminated by electrical cardioversion. Higher degree atrioventricular block (AVB) was defined as AVB > grade I. Bradyarrhythmia absoluta was defined as the presence of an irregular rhythm with fibrillatory waves and no defined P-waves, as well as heart rate < 40/min for at least 30 s. ASY was defined as the absence of electrical activity during rhythm monitoring lasting > 6 s. New-onset AF was defined as AF during ICU monitoring in the absence of AF history, as indicated by the patients´ medical record. The primary endpoint of this study was hospital mortality.

Statistical analysis was conducted using SPSS (22.0, SPSS Inc., Chicago, IL, USA) and was carried out blindly by our statistical analytic team. Descriptive statistics were obtained for all study variables. All categorical variables were compared using the chi-square test. Ordinal data were presented as the median (interquartile range (IQR)). Median values were compared using the Mann–Whitney U test. A normal distribution of continuous variables was tested using the Kolmogorov–Smirnov test. According to results, continuous variables were compared using the independent student t-test or the Mann–Whitney U test, as appropriate. Continuous data are expressed as mean and standard deviation (SD) or median (interquartile range (IQR)) values. A *p*-value < 0.05 was regarded as statistically significant.

The local ethical board of the Aerztekammer North Rhine-Westphalia stated that for this purely retrospective analysis no board approval was necessary and waived the necessity for informed consent. The study conformed with the principles outlined in the declaration of Helsinki.

## 3. Results

### 3.1. Baseline Characteristics and Outcome

Baseline characteristics and laboratory parameters are presented in Table 1. Hospital mortality was high in our collective (83.5%, Table 2). The majority of our patients were male (72.5%) and had coronary artery disease (CAD, 88.1%) as well as the associated risk factors. Median left ventricular ejection fraction was severely reduced, as assessed by echocardiography (20%). A total of 87 (79.8%) patients presented with AMI, while 61.5% of our patients received a coronary angiography and stent implantation, if adequate (Table 1).

### 3.2. Incidence of Arrhythmias during Assist Device Therapy

**Figure 1:**
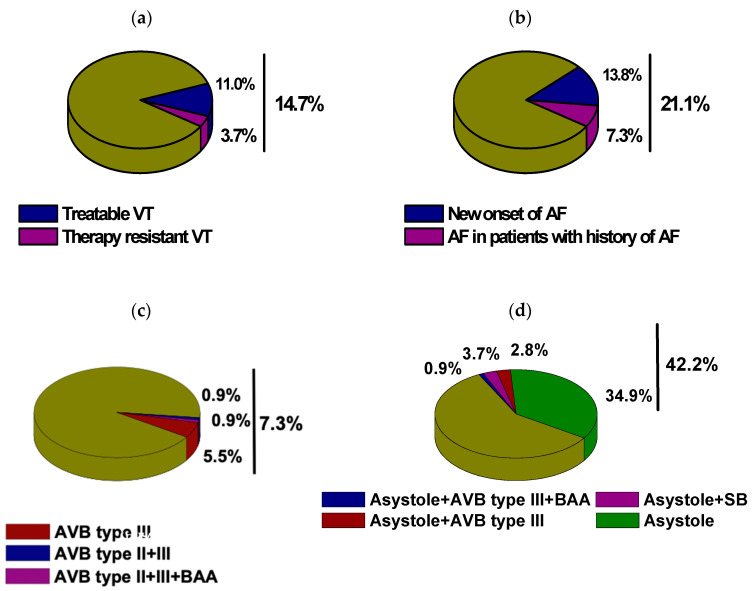
Characteristics of cardiac arrhythmias in sudden cardiac death (SCD) survivors treated with an Impella: (**a**) incidence of sustained VT, (**b**) incidence of atrial fibrillation (AF), (**c**) incidence of higher degree AV block II°/III° (AVB) type, (**d**) incidence of asystole.

In our severely ill cohort, we observed a high incidence of diverse cardiac arrhythmias, including supraventricular and ventricular tachyarrhythmias as well as bradyarrhythmias, which are specified in Table 2 and Figure 1. The incidence of atrial fibrillation (AF) was high at 21.1%, while interestingly, the majority of these cases were a new-onset of AF (13.8%, Table 2, Figure 1a). Other supraventricular tachycardias were less frequent (3.7%). Moreover, sustained ventricular tachycardias were common (14.7%); in most cases, they could be controlled by electrical cardioversion or antiarrhythmic therapy. However, 3.7% of patients developed a therapy-resistant VT. Furthermore, we found a high incidence of relevant bradyarrhythmias, which are specified in Table 2 and Figure 1c,d. In detail, we found AVB III° in 7.3% of all cases; here, 0.9% presented with AVB III + AVB II and 0.9% with AVB II° + II° + bradyarrhythmic AF (BAA). However, the majority of bradyarrhythmic cases were intermittent ASY, observed in 42.2% of our patients, indicating this to be a frequent finding in this population. Most of these potentially clinically relevant events appeared independently of other relevant bradyarrhythmias (34.9%, Table 2, Figure 1d).

### 3.3. Arrhythmias in Survivors vs. in Hospital Death

**Figure 2:**
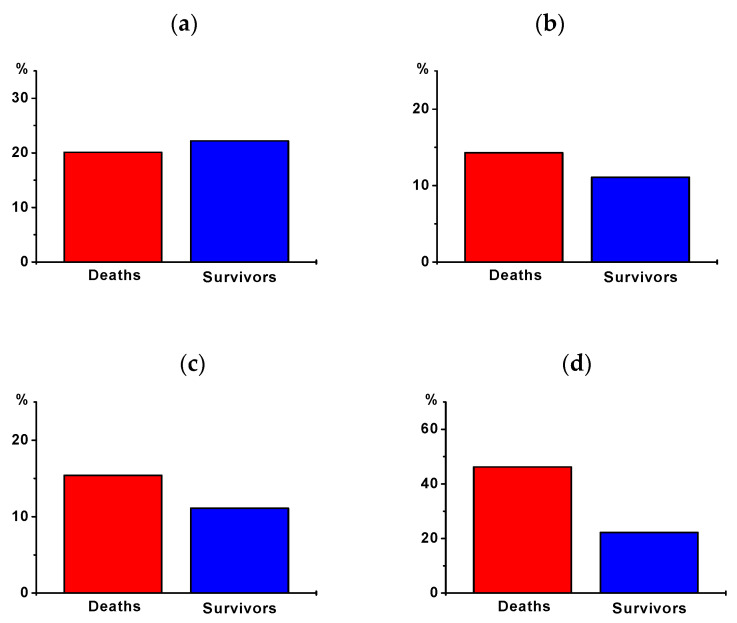
Incidence of cardiac arrhythmias in hospital death vs. survivors: (**a**) incidence of AF, (**b**) incidence of new-onset AF, (**c**) incidence of sustained VT, (**d**) incidence of asystole.

To further evaluate the potential association of the incidence of the observed cardiac arrhythmias with potentially fatal outcomes, we compared the rate of these events between hospital survivors and hospital death (Table 3, Figure 2). Despite a high incidence of AF and VT, no differences were observed between both subgroups (Table 2, Figure 2a,b). Furthermore, no association was observed when matched for the incidence of higher degree AVB (Table 2, Figure 2c). With regard to the most frequently observed cardiac arrhythmia, ASY, a trend towards a higher incidence was revealed. However, this was not statistically significant (*p* = 0.06, Table 2, Figure 2d). Four patients in our study developed a therapy-resistant VT, and all four patients, unfortunately, did not survive. One patient suffered from acute stent thrombosis of the left main coronary artery after a primary successful PCI. In the second patient, coronary revascularization failed due to an end-stage coronary three-vessel disease. The third patient, with ischemic cardiomyopathy and low output failure, developed a therapy-resistant VT despite emergency VT ablation under Impella support and various ICD therapies. The fourth patient underwent primary successful PCI but also developed hemorrhagic shock due to bleeding of the femoral vessels. The leading cause of death in our study was low output failure.

## 4. Discussion

Our study sought to analyze the incidence of cardiac arrhythmias in survivors of SCD requiring Impella support after a successful CPR. Cardiac arrhythmias were frequently observed in our collective. These included VT but also supraventricular tachyarrhythmias, mainly AF with a high rate of newly diagnosed AF. Furthermore, bradyarrhythmias were revealed with a high rate of intermittent ASY (42.2%). To the best of our knowledge, this is the first report that investigates all these pathologies in this specific collective, indicating a high arrhythmic burden in this population. Furthermore, our data reveal that the most common arrhythmic disorder observed in these patients is intermittent ASY, which could hint at a severe cardiac injury in our CPR survivors.

While ventricular arrhythmias are one of the main reasons for SCD [8], they are also frequently observed in the population of SCD survivors [9]. In the cohort of SCD survivors requiring assist device support, a further increase in arrhythmia burden can be assumed since the severity of cardiogenic shock and vasopressor therapy potentially aggravate arrhythmogenicity. This assumption is supported by the study of Le Pennec et al. [9] where 23 patients with arrhythmic storms were treated with extracorporeal life support (ECLS). Survivors frequently showed VT and ventricular fibrillation (VF) with periods of sinus rhythm, while nonsurvivors often had therapy refractory VF [9]. This issue is also underlined by the relatively high baseline lactate of 6.35 mmol/L and high mortality in our collective (83.5%, Table 1). Additionally, Le Pennec et al. showed that veno-arterial ECMO (VA-ECMO) had a positive effect on patients with incessant episodes of VT [9]. We could not observe such an effect of the Impella on therapy-resistant VT in our study, however, as this subgroup was too small to have a detectable statistical effect (*n* = 4 in our collective). Additionally, an incorrect Impella position can cause VT that can be reversed by optimizing the assist device placement [10]. However, Impella position was frequently checked in our collective and corrected when necessary. Furthermore, the majority of our patients presented with AMI and hence received an immediate coronary angiography and subsequent stent implantation in order to provide a causal treatment of SCD.

A potentially negative impact of AF on mortality in intensive care patients without an assist device has previously been described [8,11]. Therefore, we further hypothesized that cardiac arrhythmias that were commonly observed in our severely sick SCD survivors requiring Impella assistance might be a predictor of an adverse outcome. Surprisingly, neither VT, AF nor AVB correlated with mortality in our real-world collective, suggesting that the hemodynamic stabilization provided by the pump at least acutely compensated for the otherwise adverse effects of these arrhythmias. This finding is supported by Sonu et al.’s large propensity-matched study of 840 patients with cardiogenic shock requiring Impella support, which also found no impact of preexisting AF upon survival [12]. However, healthcare resource consumption as assessed by other parameters was consistently greater in patients with AF. We did not analyze these parameters in our collective since a high percentage of our patients, unfortunately, did not survive.

To the best of our knowledge, we could not find data regarding the impact of AVB in a collective similar to ours. Finally, we found ASY to be the most frequent arrhythmia in our collective. In order to exclude artifacts, e.g., due to drawing blood samples using the arterial line, we assessed the continuous ECG and the continuous arterial blood pressure simultaneously. To the best of our knowledge, we are the first group to describe the frequent occurrence of ASY in such a collective. Possibly, this is a sign of severe cardiac damage as a possible risk factor that almost reached statistical significance (*p* = 0.06). This finding is potentially explainable by other studies, e.g., that by Martinell et al. who were able to show that the absence of a rhythm other than VT/VF is a predictor of a poor outcome after out-of-hospital cardiac arrest [13]. However, this parameter did not reach statistical significance in our study and should be re-evaluated in future trials. Interestingly, asystole was significantly more frequent in patients that were treated with Impella 3.5 vs. Impella 2.5. We speculate that this result could hint at a possible negative effect of a high-performing blood pump in our collective, e.g., in “overventing” the left ventricle and possibly depriving it of the adequate preload. However, this would need to be confirmed by invasive measurements and future studies (see Supplementary Materials).

This study has several limitations due to its single-center retrospective design. Even though we were able to include 109 patients, we did not distinguish between paroxysmal, persistent and permanent atrial fibrillation and also combined the second and third degrees of AVB for statistical reasons. Additionally, we were not able to assess the impact of ablation therapy in our collective since our patients were usually too hemodynamically unstable for us to safely perform it.

## 5. Conclusions

Mortality was high in these severely sick patients. While cardiac arrhythmias were frequent, they did not predict hospital mortality in this population. The hemodynamic support of the pump seems to counterbalance the adverse effects of these events.

## Figures and Tables

**Table 1:** Baseline characteristics and laboratory parameters before assist device implantation.

Characteristics	*n*	Median (Q3–Q1) or %
Gender (female)	30	27.5%
Age	109	69.0 (18.0)
Impella 2.5 L	84	77.1%
Impella 3.5 L	25	22.9%
LVEF (%)	85	20.0 (20.00)
Acute myocardial infarction	87	79.8%
Acute PCI	67	61.5%
Duration of CPR (minutes)	105	20.00 (25.00)
Initial rhythm during CPR		
VT/VF	80	73.4%
Asystole	18	16.5%
PEA	11	10.1%
General medical history		
Arterial hypertension	62	56.9%
Diabetes mellitus	26	23.9%
Hyperlipidemia	42	38.5%
CAD	96	88.1%
PAD	9	8.3%
HFrEF	45	41.3%
HFpEF	7	6.4%
Valvular heart disease	10	9.2%
Structural heart disease	26	23.9%
Pulmonary hypertension	4	3.7%
COPD	10	9.2%
Malignancy	10	9.2%
Medical history of arrhythmias		
Atrial fibrillation	21	19.3%
Other SVT	6	5.5%
Ventricular tachycardia	27	24.8%
Laboratory		
creatinine (mg/dL)	107	1.50 (0.70)
Hemoglobin (g/dL)	107	13.00 (3.00)
Leucocytes (x/µL)	106	14,490 (10,365)
Creatine kinase (U/L)	105	320.00 (988.00)
GOT (U/L)	104	135.00 (229.25)
Lactate (U/L)	93	6.0 (6.35)
Potassium (mmol/L)	98	4.20 (0.90)

Baseline characteristics of the study population: CAD = coronary artery disease, COPD = chronic obstructive pulmonary disease, CPR = cardiopulmonary resuscitation, HFpEF = heart failure with preserved ejection fraction, HFrEF = heart failure with reduced ejection fraction, LVEF = left ventricular ejection fraction, MI = myocardial infarction, PCI = percutaneous coronary intervention, other supraventricular tachycardias = atrial flutter, atrioventricular tachycardia, atrioventricular node tachycardia or focal atrial tachycardia, PAD = periphery artery disease, PH = pulmonary hypertension, PEA = pulseless electrical activity, VF = ventricular fibrillation, VT = ventricular tachycardia.

**Table 2:** Outcomes and incidence of relevant arrhythmias during ICU monitoring of Impella therapy.

Outcomes	*n* = 109	Median (Q3–Q1) or %
General outcome		
Duration of Impella therapy (days)	104	1.00 (2.00)
ICU stay (days)	109	2.00 (4.50)
Total hospital stay (days)	109	3 (6.00)
Apoplex	4	3.7%
Total in hospital death	91	83.5%
Cause of death		
Low output failure	66	60.6%
Septic shock	15	13.8%
Hemorrhagic shock	2	1.8%
Incessant VT	4	3.7%
Brain death	2	1.8%
Abdominal ischemia or compartment	2	1.8%
Supraventricular tachycardia	25	22.9%
AF	23	21.1%
New-onset AF	15	13.8%
Other SVT	4	3.7%
Ventricular Tachycardia	20	18.3%
Non-sustained VT	5	4.6%
Sustained VT	16	14.7%
Therapy-resistant VT	4	3.7%
Bradycardia	50	45.9%
Sinus bradycardia	5	4.6%
Bradyarrhythmia absoluta	2	1.8%
Higher degree AVB	8	7.3%
Asystole	46	42.2%

AF = atrial fibrillation, AVB = atrioventricular block (higher atrioventricular block was defined as AVB > grade I), ICU = intensive care unit, SVT = supraventricular tachycardia (other SVT was defined as regular rhythm in the absence of P-waves (or appearance of flutter waves) for at least 30 s, consistent with atrial flutter, atrioventricular tachycardia, atrioventricular node tachycardia or focal atrial tachycardia), VT = ventricular tachycardia (defined as at least four consecutive ventricular beats with non-sustained ventricular tachycardia lasting < 30 s). Of note, some patients had a combination of different arrhythmias.

**Table 3:** Arrhythmia burden during Impella therapy in hospital survivals vs. hospital death.

	In Hospital Death (*n* = 91)		In Hospital Survival (*n* = 18)		*p*-Value
	*n*	%	*n*	%	
Initial rhythm during CPR					
VT/VF	64	70.3%	16	88.9%	0.103
Asystole	17	18.7%	1	5.6%	0.171
PEA	10	11.0%	1	5.6%	0.484
Arrhythmia burden during Impella therapy					
Supraventricular tachyarrhythmias					
AF	19	20.1%	4	22.2%	0.898
New-onset AF	13	14.3%	2	11.1%	0.721
Other SVT	3	3.3%	1	5.6%	0.641
Ventricular tachyarrhythmias					
Non-sustained VT	3	3.3%	2	11.1%	0.148
Sustained VT	14	15.4%	2	11.1%	0.640
Therapy-resistant VT	4	4.4%	0	0.0%	0.365
Bradyarrhythmias					
Sinus bradycardia	3	3.3%	2	11.1%	0.148
Bradyarrhythmia absoluta	2	2.2%	0	0.0%	0.526
Higher degree AVB	7	7.7%	1	5.6%	0.751
Asystole	42	46.2%	4	22.2%	0.060

AF = atrial fibrillation, AVB = atrioventricular block, Bc = bradycardia, ICU = intensive care unit, SVT = supraventricular tachycardia (other SVT was defined as regular rhythm in the absence of P-waves (or appearance of flutter waves) for at least 30 s consistent with atrial flutter, atrioventricular tachycardia, atrioventricular node tachycardia or focal atrial tachycardia), VT = ventricular tachycardia (VT was defined as at least four consecutive ventricular beats with non-sustained VT lasting < 30 s; therapy-resistant VT was defined as VT that could not be terminated by electrical cardioversion). Of note, some patients had a combination of different arrhythmias.

## Data Availability

All presented data including deidentified study participant data are available upon reasonable request to the corresponding author: Robert Larbig at contact: robert.larbig@mariahilf.de. Reuse is only permitted after agreement of all coauthors of this study.

## Supplementary Materials

The following are available online at https://www.mdpi.com/article/10.3390/jcm10071393/s1. Table S1: Characteristics of patients with atrial fibrillation vs. no atrial fibrillation, Table S2: Characteristics of patients with sustained VT vs. no sustained VT, Table S3: Characteristics of patients with asystole vs. no asystole.
