# Supplementary material for: Cardiac Arrhythmias in Survivors of Sudden Cardiac Death Requiring Impella Assist Device Therapy
Source: J Clin Med. 2021 Mar 31;10(7):1393. doi: 10.3390/jcm10071393 (PMC8037009; doi:10.3390/jcm10071393)
Supplement: Supplementary file 1 [file jcm-10-01393-s001.pdf]

Journal of
Clinical Medicine

MDPI

Supplementary Material

Table S1. Characteristics of patients with atrial fibrillation vs. no atrial fibrillation.

	n	No AF	*n*	AF	
	**86**	**Median (Q3-Q1) or %**	**23**	**Median (Q3-Q1) or %**	***p*-Value**
Gender (female)	23	26.7%	7	30.4%	0.725
Age	86	68.0 (18.0)	23	75.0 (19.0)	0.081
Impella 2.5 l	64	74.4%	20	87.0%	0.204
Impella 3.5 l	22	25.6%	3	13.0%	0.204
LVEF (%)	65	20.0 (20.0)	20	20.0 (24.0)	0.785
Duration of CPR (minutes)	83	20.00 (25.00)	22	15.00 (33.00)	0.219
Initial rhythm during CPR					
VT/VF	73	84.9%	17	73.9%	0.949
Asystole	14	16.3%	4	17.4%	0.898
PEA	9	10.5%	2	8.7%	0.802
Acute myocardial infarction	70	81.4%	17	73.9%	0.427
Acute PCI	54	62.8%	13	56.5%	0.583
**General Medical History**					
Arterial hypertension	49	57.0%	13	56.5%	0.969
Diabetes mellitus	22	25.6%	2	8.7%	0.413
Hyperlipidemia	32	37.2%	10	43.5%	0.583
CAD	76	88.4%	20	87.0%	0.852
PAD	7	8.1%	2	8.7%	0.931
HFrEF	37	43.0%	8	34.8%	0.476
HFpEF	6	7.0%	1	4.3%	0.648
Valvular heart disease	6	7.0%	4	17.4%	0.124
Structural Heart disease	21	24.4%	5	21.7%	0.789
Pulmonary hypertension	3	3.5%	1	4.3%	0.846
COPD	9	10.5%	1	4.3%	0.367
Malignancy	8	9.3%	2	8.7%	0.929
**Medical History of arrhythmias**					
Atrial fibrillation	13	15.1%	8	34.8%	0.034 *
Other SVT	3	3.5%	3	13.0%	0.074
Ventricular tachycardia	23	26.7%	4	17.4%	0.356
**Laboratory**					
Creatinine (mg/dl)	84	1.50 (1.00)	23	1.60 (1.00)	0.582
Haemoglobin (g/dl)	84	13.00 (3.00)	23	12.50 (3.00)	0.360
Leucocytes (x/µl)	83	14 000 (9 200)	23	16 860 (11 740)	0.065
Creatine kinase (U/l)	83	328.00 (1041.00)	22	264.50 (801.00)	0.420
GOT (U/l)	82	137.50 (291.00)	22	119.50 (165.00)	0.880
Lactate (U/l)	73	6.00 (6.00)	20	7.65 (8.00)	0.466
Potassium (mmol/l)	79	4.20 (0.90)	19	4.40 (1.10)	0.151

AF=atrial fibrillation, CAD= coronary artery disease, COPD= chronic obstructive pulmonary disease, CPR=cardiopulmonary resuscitation, HFpEF=heart failure with preserved ejection fraction, HFrEF=heart failure with reduced ejection fraction, LVEF=left ventricular; ejection fraction, MI=myocardial infarction, other supraventricular tachycardias = atrial flutter, atrioventricular tachycardia, atrioventricular node tachycardia or focal atrial tachycardia, PAD=periphery artery disease, PCI=percutaneous coronary intervention, PEA= Pulseless electrical, PH=pulmonary hypertension, VF=ventricular fibrillation, VT=ventricular tachycardia. * $p < 0.05$.

Table S2. Characteristics of patients with sustained VT vs. no sustained VT.

	n	No sVT	*n*	sVT	
	**93**	**Median (Q3-Q1) or %**	**16**	**Median (Q3-Q1) or %**	***p*-Value**
Gender (female)	22	23.7%	8	50.0%	0.029 *
Age	93	69.0 (19.0)	16	67.0 (13.0)	0.373
Impella 2.5 l	73	78.5%	11	68.8%	0.392
Impella 3.5 l	20	21.5%	5	31.3%	0.392
LVEF (%)	73	25.0 (23.00)	12	20.0 (23.00)	0.317
Duration of CPR (minutes)	89	21.00 (26.00)	16	10.00 (27.00)	0.068
Initial rhythm during CPR					
VT/VF	68	73.1%	12	75.0%	0.875
Asystole	15	16.1%	3	18.8%	0.794
PEA	10	10.8%	1	6.3%	0.581
Acute myocardial infarction	73	78.5%	14	87.5%	0.407
Acute PCI	57	61.3%	10	62.5%	0.927
**General Medical History**					
Arterial hypertension	52	55.9%	10	62.5%	0.623
Diabetes mellitus	22	23.7%	4	25.0%	0.907
Hyperlipidemia	35	37.6%	7	43.8%	0.642
CAD	80	86.0%	16	100.0%	0.111
PAD	8	8.6%	1	6.3%	0.752
HFrEF	38	40.9%	7	43.8%	0.828
HFpEF	5	5.4%	2	12.5%	0.283
Valvular heart disease	9	9.7%	1	6.3%	0.661
Structural Heart disease	21	22.6%	6	37.5%	0.452
Pulmonary hypertension	4	4.3%	0	0.0%	0.398
COPD	8	8.6%	2	12.5%	0.618
Malignancy	9	.%	1	6.3%	0.661
**Medical History of arrhythmias**					
Atrial fibrillation	19	20.4%	2	12.5%	0.458
Other SVT	5	5.4%	1	6.3%	0.887
Ventricular tachycardia	20	21.5%	7	43.8%	0.057
**Laboratory**					
Creatinine (mg/dl)	91	1.60 (1.00)	16	1.20 (1.00)	0.017 *
Haemoglobin (g/dl)	91	13.00 (3.00)	16	13.00 (5.00)	0.786
Leucocytes (x/µl)	91	14 250 (11 410)	15	15 460 (6 420)	0.888
Creatine kinase (U/l)	89	336.00 (984.00)	16	264.50 (2130.00)	0.530
GOT (U/l)	88	139.00 (222.00)	16	81.00 (204.00)	0.115
Lactate (U/l)	82	6.85 (6.00)	11	5.10 (4.00)	0.145
Potassium (mmol/l)	81	4.20 (0.90)	15	3.90 (0.70)	0.096

AF=atrial fibrillation, CAD= coronary artery disease, COPD= chronic obstructive pulmonary disease, CPR=cardiopulmonary resuscitation, HFpEF=heart failure with preserved ejection fraction, HFrEF=heart failure with reduced ejection fraction, LVEF=left ventricular; ejection fraction, MI=myocardial infarction, other supraventricular tachycardias = atrial flutter, atrioventricular tachycardia, atrioventricular node tachycardia or focal atrial tachycardia, PAD=periphery artery disease, PCI=percutaneous coronary intervention, PEA= Pulseless electrical, PH=pulmonary hypertension, sVT=sustained VT, VF=ventricular fibrillation, VT=ventricular tachycardia. * $p < 0.05$.

Table S3. Characteristics of patients with asystole vs. no asystole.

	n	No Asystole	*n*	Asystole	
	**63**	**Median (Q3-Q1) or %**	**46**	**Median (Q3-Q1) or %**	***p*-Value**
Gender (female)	21	33.3%	9	19.6%	0.112
Age	63	69.0 (20.0)	46	69.5 (18.0)	0.454
Impella 2.5 l	54	85.7%	30	65.2%	0.012*
Impella 3.5 l	9	14.3%	16	34.8%	0.012*
LVEF (%)	49	25.0 (25.00)	36	20.0 (20.00)	0.241
Duration of CPR (minutes)	61	22.00 (28.00)	44	17.50 (25.00)	0.438
Initial rhythm during CPR					
VT/VF	47	74.6%	33	71.7%	0.738
Asystole	9	14.3%	9	19.6%	0.463
PEA	7	11.1%	4	15.2%	0.679
Acute myocardial infarction	52	82.5%	35	76.1%	0.407
Acute PCI	39	61.9%	28	60.9%	0.913
**General Medical History**					
Arterial hypertension	36	57.1%	26	56.5%	0.948
Diabetes mellitus	13	20.6%	13	28.3%	0.356
Hyperlipidemia	26	41.3%	16	34.8%	0.492
CAD	56	88.9%	40	87.0%	0.759
PAD	6	9.5%	3	6.5%	0.574
HFrEF	26	41.3%	19	41.3%	0.997
HFpEF	5	7.9%	2	4.3%	0.450
Valvular heart disease	5	7.9%	5	10.9%	0.600
Structural Heart disease	18	28.6%	8	17.4%	0.176
Pulmonary hypertension	3	4.8%	1	2.2%	0.478
COPD	4	6.3%	6	13.0%	0.232
Malignancy	6	9.5%	4	8.7%	0.882
**Medical History of arrhythmias**					
Atrial fibrillation	10	15.9%	11	23.9%	0.293
Other SVT	2	3.2%	6	13.0%	0.212
Ventricular tachycardia	17	27.0%	10	21.7%	0.531
**Laboratory**					
Creatinine (mg/dl)	61	1.50 (1.00)	46	1.70 (1.00)	0.264
Haemoglobin (g/dl)	61	13.00 (3.00)	46	13.55 (4.00)	0.313
Leucocytes (x/µl)	61	12 790 (10 905)	45	16 900 (9 780)	0.153
Creatine kinase (U/l)	60	310.50 (1121.00)	45	336.00 (750.00)	0.424
GOT (U/l)	60	153.00 (287.00)	44	113.50 (202.00)	0.172
Lactate (U/l)	53	7.10 (6.00)	40	5.75 (7.00)	0.566
Potassium (mmol/l)	57	4.29 (0.70)	41	4.20 (1.00)	0.694

AF=atrial fibrillation, CAD= coronary artery disease, COPD= chronic obstructive pulmonary disease, CPR=cardiopulmonary resuscitation, HFpEF=heart failure with preserved ejection fraction, HFrEF=heart failure with reduced ejection fraction, LVEF=left ventricular; ejection fraction, MI=myocardial infarction, other supraventricular tachycardias = atrial flutter, atrioventricular tachycardia, atrioventricular node tachycardia or focal atrial tachycardia, PAD=periphery artery disease, PCI=percutaneous coronary intervention, PEA= Pulseless electrical, PH=pulmonary hypertension, sVT=sustained VT, VF=ventricular fibrillation, VT=ventricular tachycardia. * $p < 0.05$.
